# Supplementary material for: Pathogen reduction co-benefits of nutrient best management practices
Source: PeerJ. 2016 Nov 22;4:e2713. doi: 10.7717/peerj.2713 (PMC5126620; doi:10.7717/peerj.2713)
Supplement: Supplemental Information 1 [file peerj-04-2713-s001.docx]

Table A-2. Total Loading Reduction Estimates for the Potomac River Basin.

| **BMPS** | **Land Use** | **BMP acres Phase II - Potomac** | **% Land use category covered by BMP** | **Average FIB reduction** | **Weighted sum (efficiency * % BMP cover)** | **Potential reduction at edge of stream (cfu/yr)** | **Potential reduction main channel (cfu/yr)** | **% Loadings Reduced** |
| --- | --- | --- | --- | --- | --- | --- | --- | --- |
| ***Pasture Practices*** |  |  |  |  |  |  |  |  |
| Barnyard Runoff Control | Pasture | 3,028 | 0.33% | 81% | 0.0027 |  |  |  |
| Loafing Lot Management | Pasture | 55 | 0.01% | 75% | 0.0000 |  |  |  |
| Pasture Alternative Watering | Pasture | 20,702 | 2.25% | 90% | 0.0202 |  |  |  |
| Prescribed Grazing | Pasture | 165,042 | 17.92% | 80% | 0.1425 |  |  |  |
| Precision Intensive Rotational Grazing | Pasture | 31,017 | 3.37% | 90% | 0.0303 |  |  |  |
| Horse Pasture Management | Pasture | 15,074 | 1.64% | 72% | 0.0118 |  |  |  |
| Forest Buffers on Fenced Pasture Corridor | Pasture | 3,845 | 0.42% | 50% | 0.0021 |  |  |  |
| Grass Buffers on Fenced Pasture Corridor | Pasture | 6,741 | 0.73% | 77% | 0.0056 |  |  |  |
| Stream Access Control with Fencing | Pasture | 27,919 | 3% | 36% | 0.0108 |  |  |  |
| **Total Pasture Reduction (pasture + feedlots)** | | 273,423 | **30%** |  | 0.2261 | **8.07E+16** | **1.73E+16** | **23%** |
| ***Agriculture Practices*** |  |  |  |  |  |  |  |  |
| Forest Buffers | Crop | 41,934 | 2.98% | 43% | 0.0128 |  |  |  |
| Wetland Restoration | Crop | 13,156 | 0.94% | 35% | 0.0033 |  |  |  |
| Land Retirement | Crop | 39,312 | 2.80% | 93% | 0.0260 |  |  |  |
| Grass Buffers | Crop | 41,700 | 2.97% | 69% | 0.0205 |  |  |  |
| Water Control Structures | Crop | 238 | 0% | 67% | 0.0001 |  |  |  |
| **Total Crop Reduction** |  | 136,341 | **10%** |  | 0.0627 | **4.57E+15** | **9.80E+14** | **6%** |
| ***Urban/Suburban Practices*** |  |  |  |  |  |  |  |  |
| Wet Ponds & Wetlands | Urban | -9,098 | -0.7% | 48% | -0.0035 |  |  |  |
| Dry Ponds | Urban | -78,767 | -6.3% | 80% | -0.0506 |  |  |  |
| Extended Dry Ponds | Urban | -6,324 | -0.5% | 80% | -0.0041 |  |  |  |
| Infiltration Practices | Urban | 69,533 | 5.6% | 93% | 0.0519 |  |  |  |
| Filtering Practices | Urban | 112,630 | 9.0% | 75% | 0.0678 |  |  |  |
| BioRetention | Urban | 15,321 | 1.2% | 71% | 0.0087 |  |  |  |
| BioSwale | Urban | 6,685 | 0.5% | -6% | -0.0003 |  |  |  |
| Retrofit Stormwater Management | Urban | 354 | 0.0% | 57% | 0.0002 |  |  |  |
| Erosion and Sediment Control | Urban | -29,738 | -2.4% | 57% | -0.0135 |  |  |  |
| Impervious Surface Reduction | Urban | 21,904 | 1.8% | 57% | 0.0099 |  |  |  |
| Forest Buffers | Urban | 12,177 | 1% | 43% | 0.0042 |  |  |  |
| **Total Urban Reduction (urban + septic)** |  | 114,676 | **9%** |  | 0.0708 | **1.60E+15** | **3.44E+14** | **7%** |
| **Potomac River Basin Total (all sources)** |  |  |  |  |  | **8.69E+16** | **1.86E+16** | **19%** |
