# Supplementary material for: Pathogen reduction co-benefits of nutrient best management practices
Source: PeerJ. 2016 Nov 22;4:e2713. doi: 10.7717/peerj.2713 (PMC5126620; doi:10.7717/peerj.2713)
Supplement: Supplemental Information 2 [file peerj-04-2713-s002.docx]

Table A-3. Total Loading Reduction Estimates for the Chesapeake Bay Watershed.

| **BMPS** | **Land Use** | **BMP acres Phase II - Potomac** | **% of land use category covered by BMP** | **Average FIB reduction** | **Weighted sum (efficiency * % BMP cover)** | **Potential reduction at edge of stream (cfu/yr)** | **Potential reduction main channel (cfu/yr)** | **% Loadings Reduced (of delivered to tidal)** |
| --- | --- | --- | --- | --- | --- | --- | --- | --- |
| ***Pasture Practices*** |  |  |  |  |  |  |  |  |
| Barnyard Runoff Control | Pasture | 12,055 | 0.49% | 81% | 0.0040 |  |  |  |
| Loafing Lot Management | Pasture | 498 | 0.02% | 75% | 0.0002 |  |  |  |
| Pasture Alternative Watering | Pasture | 83,693 | 3.43% | 90% | 0.0309 |  |  |  |
| Prescribed Grazing | Pasture | 545,282 | 22.36% | 80% | 0.1778 |  |  |  |
| Precision Intensive Rotational Grazing | Pasture | 277,657 | 11.39% | 90% | 0.1025 |  |  |  |
| Horse Pasture Management | Pasture | 81,062 | 3.32% | 72% | 0.0239 |  |  |  |
| Forest Buffers on Fenced Pasture Corridor | Pasture | 13,395 | 0.55% | 50% | 0.0027 |  |  |  |
| Grass Buffers on Fenced Pasture Corridor | Pasture | 24,217 | 0.99% | 77% | 0.0076 |  |  |  |
| Stream Access Control with Fencing | Pasture | 60,807 | 2% | 36% | 0.0089 |  |  |  |
| **Total Pasture Reduction (pasture + feedlots)** | | 1,098,666 | **45%** | 0 | 0.3585 | **3.39E+17** | **7.28E+16** | **36%** |
| ***Agriculture Practices*** |  |  |  |  |  |  |  |  |
| Forest Buffers | Crop | 202,951 | 3.06% | 43% | 0.0131 |  |  |  |
| Wetland Restoration | Crop | 86,978 | 1.31% | 35% | 0.0046 |  |  |  |
| Land Retirement | Crop | 328,392 | 4.95% | 93% | 0.0460 |  |  |  |
| Grass Buffers | Crop | 173,492 | 2.61% | 69% | 0.0180 |  |  |  |
| Water Control Structures | Crop | 28,616 | 0% | 67% | 0.0029 |  |  |  |
| **Total Crop Reduction** |  | 820,429 | **12%** |  | 0.0846 | **2.91E+16** | **6.25E+15** | **8%** |
| ***Urban/Suburban Practices*** |  |  |  |  |  |  |  |  |
| Wet Ponds & Wetlands | Urban | 98,290 | 2.0% | 48% | 0.0097 |  |  |  |
| Dry Ponds | Urban | -452,870 | -9.3% | 80% | -0.0747 |  |  |  |
| Extended Dry Ponds | Urban | 11,289 | 0.2% | 80% | 0.0019 |  |  |  |
| Infiltration Practices | Urban | 545,939 | 11.2% | 93% | 0.1046 |  |  |  |
| Filtering Practices | Urban | 740,706 | 15.3% | 75% | 0.1145 |  |  |  |
| BioRetention | Urban | 47,980 | 1.0% | 71% | 0.0070 |  |  |  |
| BioSwale | Urban | 13,142 | 0.3% | -6% | -0.0001 |  |  |  |
| Retrofit Stormwater Management | Urban | 24,513 | 0.5% | 57% | 0.0029 |  |  |  |
| Erosion and Sediment Control | Urban | -56,349 | -1.2% | 57% | -0.0066 |  |  |  |
| Impervious Surface Reduction | Urban | 61,683 | 1.3% | 57% | 0.0072 |  |  |  |
| Forest Buffers | Urban | 37,454 | 1% | 43% | 0.0033 |  |  |  |
| **Total Urban Reduction (urban + septic)** |  | 1,071,777 | **22%** |  | 0.1697 | **1.50E+16** | **3.21E+15** | **17%** |
| **Chesapeake Basin Total (all sources)** |  |  |  |  |  | **3.83E+17** | **8.22E+16** | **27%** |
